# Influence of Al/Ti Ratio and Ta Concentration on the As-Cast Microstructure, Phase Composition, and Phase Transformation Temperatures of Lost-Wax Ni-Based Superalloy Castings

**DOI:** 10.3390/ma15093296

**Published:** 2022-05-04

**Authors:** Małgorzata Grudzień-Rakoczy, Łukasz Rakoczy, Rafał Cygan, Konrad Chrzan, Ondrej Milkovič, Zenon Pirowski

**Affiliations:** 1 Łukasiewicz Research Network-Krakow Institute of Technology, Zakopianska 73, 30-418 Krakow, Poland; konrad.chrzan@kit.lukasiewicz.gov.pl (K.C.); zenon.pirowski@kit.lukasiewicz.gov.pl (Z.P.); 2 Faculty of Metals Engineering and Industrial Computer Science, AGH University of Science and Technology, al. Mickiewicza 30, 30-059 Krakow, Poland; lrakoczy@agh.edu.pl; 3 Consolidated Precision Products Corporation, Investment Casting Division, Hetmańska 120, 35-078 Rzeszow, Poland; rafal.cygan@cppcorp.com; 4 Institute of Materials Research, Slovak Academy of Sciences, Watsonova 47, 040 01 Košice, Slovakia; omilkovic@saske.sk; 5 Institute of Experimental Physics, Slovak Academy of Sciences, Watsonova 47, 040 01 Košice, Slovakia

**Keywords:** Inconel 740, phase transformation, investment casting, superalloy, solidification

## Abstract

The as-cast microstructure, alloying element segregation, solidification behavior, and thermal stability of model superalloys based on Inconel 740 with various Al/Ti ratios (0.7, 1.5, 3.4) and Ta (2.0, 3.0, 4.0 wt%) concentrations were investigated via ThermoCalc simulations, scanning and transmission electron microscopy, energy-dispersive X-ray spectroscopy, dilatometry, and differential scanning calorimetry. The solidification of the superalloys began with the formation of primary γ dendrites, followed by MC carbides. The type of subsequently formed phases depended on the superalloys’ initial Al/Ti ratio and Ta concentration. The results obtained from solidification simulations were compared to the obtained microstructures. For all castings, the dendritic regions consisted of fine γ′ precipitates, with their size mainly depending on the initial Al/Ti ratio, whereas in the interdendritic spaces, (Nb, Ta, Ti)C carbides and Nb-rich Laves phase precipitates were present. In high Al/Ti ratio superalloys, β-NiAl precipitates, strengthened by η and α-Cr phases, were observed. Based on dilatometric results, the dissolution of γ′ precipitates was accompanied by a substantial increase in the coefficient of thermal expansion. The end of the dilatation effect took place around the γ′ solvus temperature, as determined via calorimetry. Moreover, the bulk solidus temperature was preceded by the dissolution of the Laves phase, which may be accompanied by local melting.

## 1. Introduction

Ni-based superalloys are a class of materials extensively used for hot components in industrial power plants and aerospace engines, due to their excellent creep strength, oxidation, and hot-corrosion resistance at elevated service temperatures [1]. Advanced ultra-supercritical (A-USC) power plant designs are expected to have efficiencies of coal-fired power plants above 35 pct. With component service temperatures approaching 760 °C, Ni-based superalloys must replace ferritic-martensitic, high-strength steels, and austenitic stainless steels to fulfill the long-term, high-temperature strength requirements in these pressurized cycles [2,3]. The IN740 Ni-based superalloy has been studied within the scope of European projects leading to COM-TES700 test loop exposures at the Esbjerg (Sweden) and Scholven (Germany) power plants [4]. IN740 was developed on the basis of the Nimonic 263 superalloy chemical composition [5]. Compared to Nimonic 263, Cr and Mo concentrations in IN740 were optimized to achieve improved sulfidation resistance, whereas Nb was added to increase high-temperature strength [6]. The US A-USC Consortium applies higher service temperatures (760 °C) that require steam transfer pipes and boiler tubing to be produced from precipitation-strengthened alloys with a higher volume fraction of intermetallic phase precipitates. Up to 725 °C, IN740 exhibits good microstructural stability during long-term annealing. At 760 °C, however, three main limitations appear, i.e., the high coarsening rate of the γ′ phase, plate-like η-phase formation, and high Si-containing brittle G-phase formation, which lead to a decrease in strength and ductility [7,8,9]. Modifying the IN740 Ni-based superalloy’s chemical composition has been the subject of several research studies. Shin et al. [10] investigated the influence of Mo (0.5, 3.0, 6.0 wt.%) on the γ′ precipitates’ coarsening kinetics in IN740. The authors found that the coarsening rate of the γ′ precipitates decreased with increasing Mo, and the activation energies for coarsening were 245 kJ/mol, 261 kJ/mol, and 278 kJ/mol for 0.5, 3.0, and 6.0 wt.% Mo, respectively. Abbasi et al. [11] investigated the influence of Al and Ti concentrations on IN740′s microstructure stability and oxidation resistance. It was shown that an increase in Al concentration (up to 2.4 wt.%), with low Ti contents, leads to grain refinement, improves oxidation resistance, and reduces the number of voids in the vicinity of the superalloy/oxide layer boundary. The opposite effect, however, was found for increased Ti concentrations. The result was an increased mass gain after oxidation and numerous voids at the superalloy/oxide layer boundary, due to TiO_2_ precipitates not constituting a strong enough barrier for Cr and O ions. The most popular and commercially used IN740 superalloy modification is the IN740H superalloy, which satisfies the service requirements for A-USC applications [12]. Presently, IN740H is widely used in A-USC power plants in wrought form as boiler components. However, large complex components in boilers and other casings in gas turbines and valve chests additionally require thick-wall castings. Using the material in its cast variant would be highly beneficial in terms of size, geometry, and complexity range [13,14]. Detrois et al. [15,16] have indicated possible ductility losses during creep in castings, with a chemical composition in line with IN740′s composition. They suggested that excessive carbides at the grain boundaries are responsible for the effect. Despite the presence of Cr-rich M_23_C_6_ carbides, which impede grain-boundary sliding by restricting deformation at the boundaries, the local chemistry can change and subsequently lead to the formation of cracking initiation sites. In this work, IN740 superalloy chemical composition modifications consisted of adding Al (increase in Al/Ti ratio) as the main γ′-former and introducing Ta, which is widely known as a strong MC-carbide-former and limits the formation of M_23_C_6_ carbides [17,18]. The reported order of most to least stable MC carbide is TaC > NbC > TiC > VC, where Nb is approximately equivalent to tantalum in stabilizing MC carbides [19]. As the production of superalloys in the form of large castings is desirable, the process was carried out in an argon gas atmosphere, rather than in vacuum. The casting of Ni-based superalloys presents unique challenges, as the microstructure segregates during casting. The casting process of Ni-based superalloys, with a high refractory element content, is usually followed by solution heat treatment, which can also be challenging [20]. As the diffusivity of the alloying elements is low and the diffusion path from dendrite cores to interdendritic spaces is long, it is difficult to homogenize the chemical composition via heat treatment. Furthermore, due to the remaining eutectic phases in the interdendritic spaces, the local solidus temperature is lower than the bulk solidus temperature [21]. If the solutioning temperature is not carefully selected, incipient melting will take place. In this sense, the solutioning of Ni-based superalloys by means of conventional processing requires a long time. Well-designed heating scenarios will be needed to mitigate the risk of incipient melting and achieve as much chemical homogenization as possible [22,23]. Analyzing the material’s solidification behavior, phase composition, and stability is necessary to prepare the best procedure to dissolve those as much as possible during the solution heat treatment, without risk of incipient melting of the superalloy. The results from this work could provide significant insight into composition optimization and solutioning heat treatment control of novel Ni-based superalloys.

## 2. Materials and Methods

### 2.1. Materials

**Figure 1:**
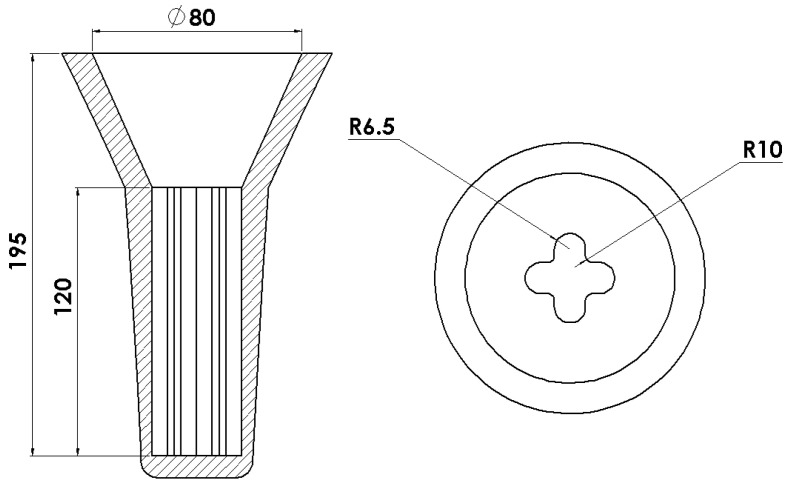
The geometry of the shell mold used for the castings preparation.

For the purposes of this work, nine Ni-based superalloys, with increased Al concentration and Ta addition compared to the standard IN740, were cast. The castings were manufactured via lost-wax casting. First, wax models, combined with a gating system, were prepared (Figure 1).

The chemical composition modification procedures for the nine cast Ni-based superalloys are presented in Table 1. A 3 kg IN740 ingot was inductively melted in a corundum crucible for 35 min in an argon atmosphere. Next, the alloying additions of Al (rods with diameter 6 mm and length 100 mm, 99.99% purity) and Ta (disks ∅10 × 0.2 mm, 99.9% purity) were added. The melted superalloy was then deoxygenated twice using a Ni-Mg compound (before melt-pouring). The pouring temperature was in the range of 1500–1550 °C, and was controlled by S-type thermocouples (PtRh10-Pt), with a ∅ 0.8 mm wire used for temperature measurements. For more detailed instructions on casting procedures and shell mold fabrication, please consult our previous paper [24]. The chemical compositions of all investigated materials, given in Table 2, were analyzed using a spark optical emission spectrometer.

### 2.2. Methods

The Thermo-Calc software (version 2021a, Stockholm, Sweden), with the TCNI10 database, was used to analyze the solidification process using the Scheil model. The Scheil model considers the segregation of solute elements during solidification and assumes the absence of back-diffusion during calculations. Complete solidification is assumed with less than 1% liquid present in the calculation, and generally, the solidification range is quite broad [25]. In this work, the model was used to predict the type of primary phases in the as-cast superalloys. The superalloys were prepared into 10 × 10 × 5 mm samples and their surface was mechanically polished. The samples were then subjected to X-ray diffraction (XRD) to identify the formed phases. Measurements were made in Bragg–Brentano geometry using a Philips X’Pert Pro MRD diffractometer, (Philips, Amsterdam, The Netherlands) equipped with Cu radiation (λ = 1.54 Å). Measurements were made at room temperature in the range of 2ϴ = 30–100°, with a step size of 0.01671°.

For scanning electron microscopy (SEM) characterization, specimens (10 × 10 × 5 mm) were cut from the middle of each casting and mounted in resin, ground, polished, and electrochemically etched in 10% oxalic acid. Back-scattered electron (BSE) imaging and energy-dispersive X-ray spectroscopy (EDX) were performed using 20 kV accelerating voltage, set on a Phenom XL (Phenom-World, Eindhoven, The Netherlands) microscope, equipped with an EDX detector. Semi-quantitative chemical analyses were carried out using the ZAF correction. Observations of γ′ precipitates in dendritic regions were performed using a high-resolution Merlin Gemini II microscope (ZEISS, Oberkochen, Germany), equipped with a Schottky field-emission gun (Bruker, Billerica, MA, USA). The images were obtained using secondary electrons (SE).

Quantitative analysis of the γ′ precipitates and carbides (manually contoured on images) was carried out using ImageJ (National Institutes and the Laboratory for Optical and Computational Instrumentation, University of Wisconsin, Madison, WI, USA). The measurements were conducted in five places on each casting, which included more than 200 γ′ precipitates (SEM-SE, magnification ×100,000) and at least 50 carbide precipitates (SEM-BSE, magnification ×10,000). Next, the precipitates’ area (A) and perimeter (P) were determined. The size of γ′ precipitates in the dendritic regions and carbides in the interdendritic spaces were represented as the equivalent diameter (Equation (1)), while the circularity of γ′ precipitates was calculated to evaluate their morphological differences (Equation (2)).

(1) $$\bar{\;\phi }=\sqrt{\frac{4A}{\pi }}$$

(2) $$\zeta =\frac{4\pi A}{P^{2}}$$

Samples A3, A6, and A9 were investigated in more detail via TEM using bright field (BF), selected area electron diffraction (SAED), and high-angle annular dark-field scanning transmission electron microscopy (HAADF-STEM) methods. Samples were first mechanically ground down to a thickness of about 0.05 mm, after which 3 mm discs were punched and dimpled using a Gatan dimple grinder (Gatan, Inc., Pleasanton, CA, USA) on both sides. The final step was thinning using an Ar^+^ ion beam (PIPS of Gatan) at low angles. Before loading into the microscope, the thin foils were plasma-cleaned (NanoMill 1040 Fischione) to remove surface contaminations. Observations of samples A3 and A6 were performed using a Tecnai G2 20 TWIN (FEI) microscope. Analysis of the A9 superalloy was carried out with the Cs-corrected probe FEI Titan^3^ G2 60–300 TEM with the ChemiSTEM system (Thermo Fisher Scientific, Eindhoven, The Netherlands). The accelerating voltage was 300 kV. The STEM-EDX data were acquired at 300 kV and were then subjected to analysis using the Esprit software (Bruker, v1.9) (Bruker, Billerica, MA, USA), in which the standardless Cliff–Lorimer quantification method was applied. The JEMS software (JEMS-SWISS, Jongny, Switzerland) was applied to fit the diffraction peaks. To analyze the phase transformations in the elevated temperatures, dilatometry and differential scanning calorimetry were carried out. The dilatometry measurements were carried out with a NETZSCH DIL 402C/4/G (Netzsch, Selb, Germany) device on samples ϕ6 × 25 mm up to melting point (under argon shielding) with a heating rate of 0.08 °C/s. Differential scanning calorimetry was performed using a Pegasus DSC 404C/3/G (Netzsch, Selb, Germany) device on samples weighing about 40 mg in the range 25–1480 °C with a heating rate of 10 °C/min. 

## 3. Results

### 3.1. Solidification Simulation via Thermo-Calc

**Figure 2:**
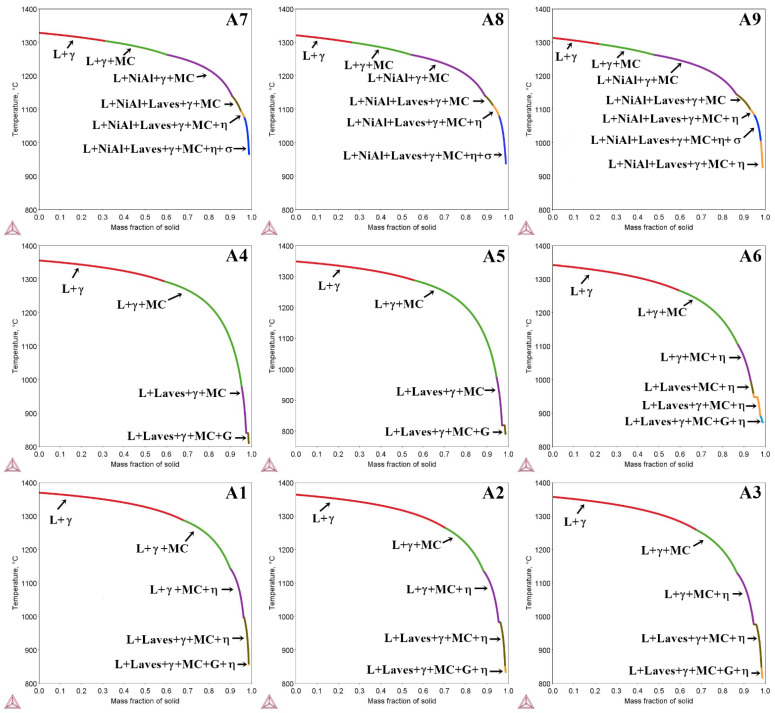
Scheil solidification simulation of the (A1–A9) superalloys.

Figure 2 presents the solidification process predictions of the A1–A9 superalloys, based on Scheil’s model. The solidification process of low Al/Ti ratio superalloys, with Ta additions in the range of 2.0 to 4.0 wt%, is predicted to begin with γ phase crystal formation at 1369 °C (A1), 1364 °C (A2), and 1355 °C (A3), followed by MC carbide precipitation at 1289 °C (A1), 1266 °C (A2), and 1260 °C (A3). Upon further temperature decrease, and at the stage where the mass fraction of the solid exceeds 0.9, the Laves phase is expected. Finally, the Scheil model predicts the formation of the G phase in the final stage of solidification. In the case of superalloys A4, A5, and A6 (medium Al/Ti ratio), solidification with γ phase formation is estimated to begin at 1355 °C, 1349 °C, and 1341 °C, respectively, whereas MC carbides are expected to precipitate at 1294 °C, 1288 °C, and 1267 °C. Additionally, superalloys A3 and A4 share the same liquidus temperature. The data show that for constant Ta content, an increase in Al/Ti ratio leads to an increase in the MC carbide precipitation temperature. At 1106 °C, the precipitation of the η phase is predicted in superalloy A6. The lack of η phase in the A4 and A5 superalloys indicates that sufficiently high Ta concentration at the medium Al/Ti ratio is required for its formation. All superalloys are projected to contain Laves phase precipitations. Similarly to superalloys with a low Al/Ti ratio, the model predicts G phase formation in the final solidification stage for medium Al/Ti ratios. The results of the Scheil solidification simulation of superalloys A7, A8, and A9 indicate that their liquidus temperature is the lowest of all tested superalloys, i.e., 1328 °C, 1320 °C, and 1313 °C, respectively. As in the case of superalloys with low and medium Al/Ti ratios, an increase in Ta concentration is accompanied by a decrease in the liquidus temperature. The MC carbides are speculated to precipitate at higher temperatures, when the mass fraction of the liquid phase is higher than that of the solid phase. At 1263 °C, the β-NiAl phase is expected to form, regardless of the Ta concentration. With further cooling, the Laves phase should precipitate at 1139 °C (A7), 1141 °C (A8), and 1145 °C (A9). The η phase is estimated to precipitate at 1093 °C in superalloy A7, whereas for samples A8 and A9, these temperatures are expected to be slightly higher, at 1100 °C and 1101 °C, respectively. The last phase predicted to form from the liquid is the σ phase. Unlike in castings A1–A6, no G phase is expected.

### 3.2. Analysis of the Microstructure of the A1–A9 Superalloys in As-Cast Condition 

**Figure 3:**
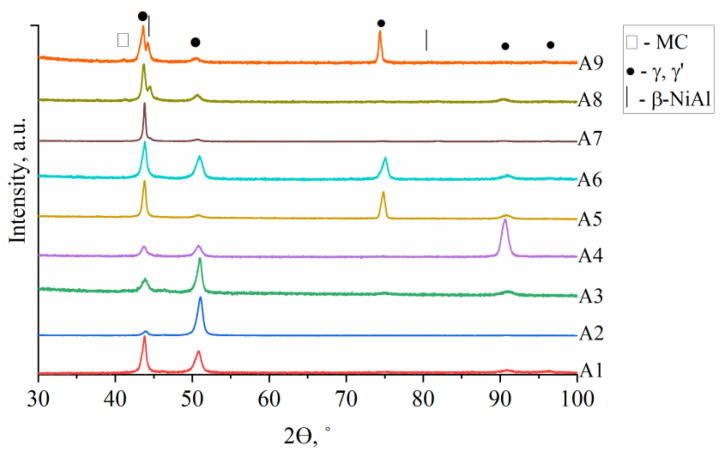
XRD spectra of the as-cast A1–A9 superalloys.

The XRD patterns of the as-cast superalloys are presented in Figure 3. Peaks of four phases were identified, namely the γ matrix, γ′ intermetallic phase, β-NiAl, and MC carbides. 

**Figure 4:**
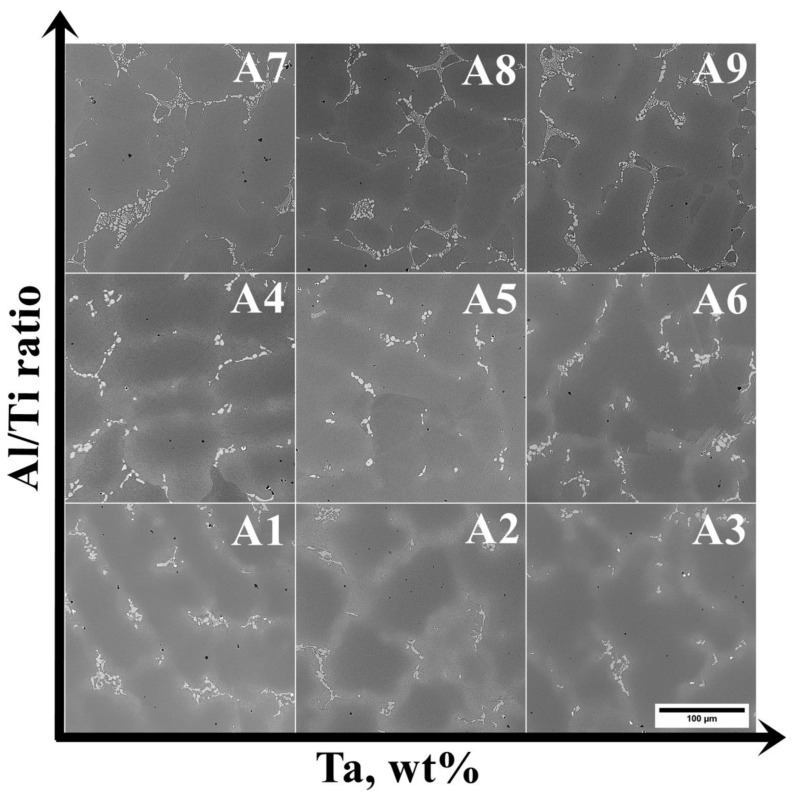
Dendritic structure of the as-cast (A1–A9) superalloys, SEM-BSE.

The microstructures of the as-cast A1–A9 superalloys are shown in Figure 4. The dendritic regions are characterized by a relatively homogenous microstructure, whereas precipitates with bright phase contrast can be found in the interdendritic regions. The highest percentage of precipitates in the interdendritic spaces is observed in the A7–A9 superalloys. Local fine precipitates with a black contrast are visible in all castings. 

**Figure 5:**
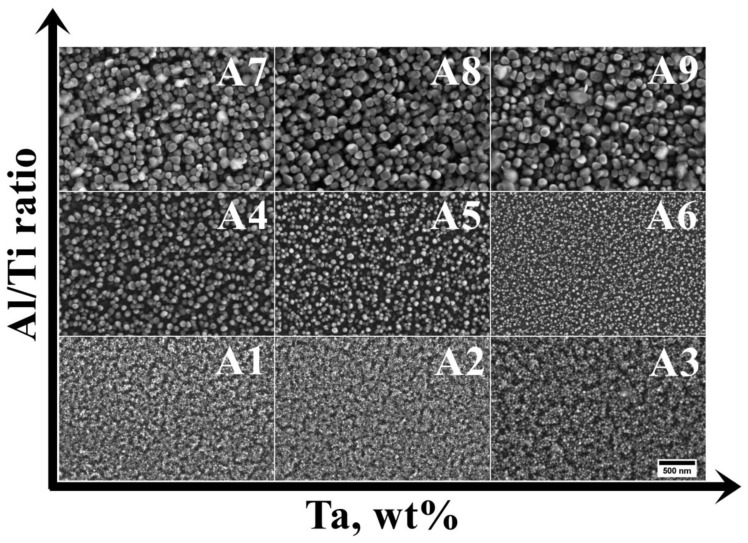
Precipitates in the dendritic regions of the (A1–A9) castings, SEM-SE.

The dendritic regions, regardless of the casting variant, are predominantly composed of very fine precipitates with a spherical or near-cubic morphology (Figure 5). The finest precipitates were observed in variants A1–A3 (low Al/Ti ratio), while the largest were observed in samples with the highest Al/Ti ratio. These precipitates were subjected to stereological parameter analyses, and the results are summarized in Table 3.

In superalloys with low and high Al/Ti ratios, the mean size of the γ′ precipitates increases with Ta concentration. The mean diameter increases from 26 nm to 46 nm in the first group, while in the second group, it increases from 110 nm to 134 nm. In superalloys with a medium Al/Ti ratio, a decrease in the mean size of the γ′ precipitates from 99 nm to 41 nm with increasing Ta is observed. 

**Figure 6:**
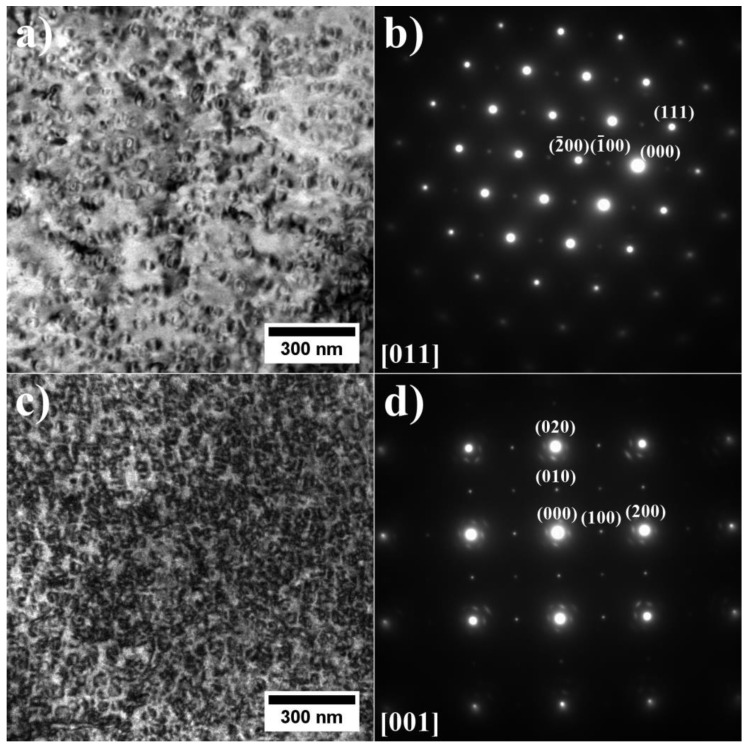
Morphology of the γ′ precipitates in the dendritic region and corresponding SAED patterns: (**a**,**b**) A3; (**c**,**d**) A6, TEM-BF.

The precipitates in the dendrites were analyzed by TEM and SAED (Figure 6). It was confirmed that the γ matrix is strengthened by coherent γ′ precipitates. Since the lattice parameters of the disordered matrix and ordered γ′ precipitates are very similar, the electron diffraction patterns exhibit some common maxima, e.g., {200} reflections. Additionally, {100} superlattice reflections, originating only from γ′ precipitates, are also present. The crystallographic relationship is as follows: {100} γ//{100} γ′; <010> γ//<010> γ′, which is referred to as the cube-cube orientation relationship. 

**Figure 7:**
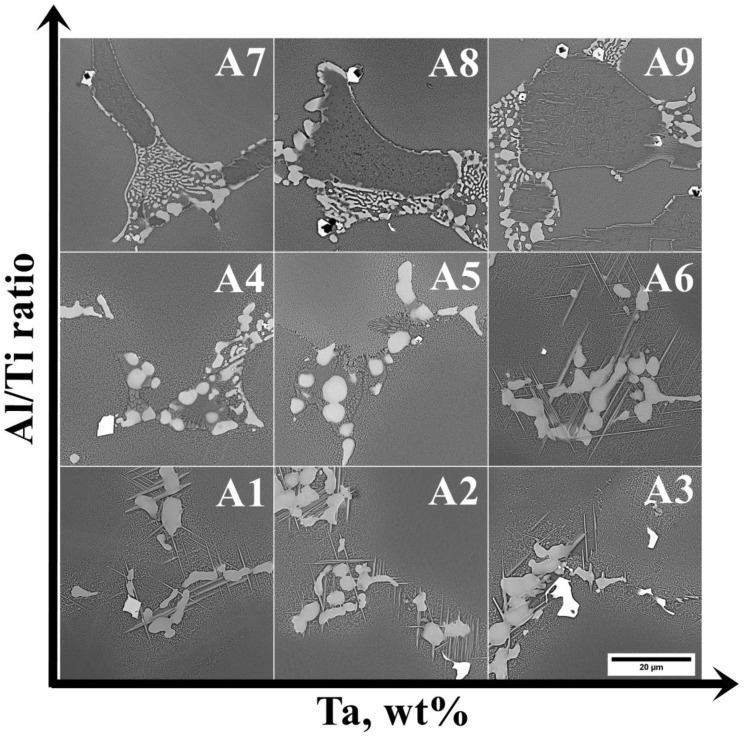
Morphology of precipitates in the interdendritic spaces of the (A1–A9) castings, SEM-BSE.

In the interdendritic spaces, the microstructure of the castings is more complex. Numerous precipitates of various distributions, geometries, sizes, and phase contrasts are observed (Figure 7). 

**Figure 8:**
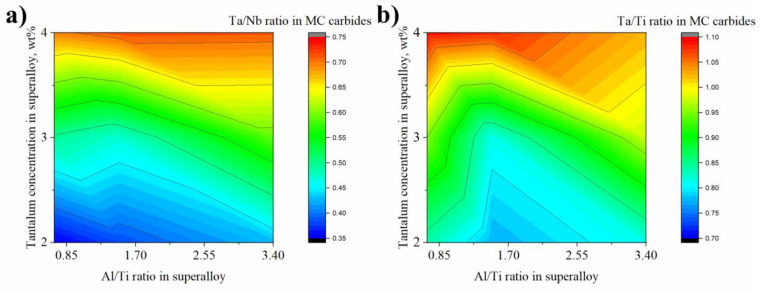
The concentration relationship in the MC carbides: (**a**) Ta/Nb; (**b**) Ta/Ti, calculated based on the semi-quantitative SEM-EDX measurements.

MC carbides (bright precipitates: high Z-number) are considered to be the second most important precipitates, after γ′ precipitates, in strengthening polycrystalline Ni-based superalloys. Semi-quantitative SEM-EDX analyses were carried out and it was observed that the carbides are composed mainly of three elements: Nb, Ti, and Ta (Figure 8). Based on Ta/Nb atomic concentration relationship calculations, it can be observed that the increase in Ta concentration independently of the Al/Ti ratio leads to the partial replacement of Nb in the MC carbides composition (Figure 8a). It looks visible with increasing Ta contents in the superalloy. A similar effect is observed for the Ta/Ti relationship (Figure 8b), where an increase in Ta contents led to an increase in the Ta/Ti ratio in MC carbides. Based on these results, the main carbide-alloying element was found to be Nb. Whether Ta or Ti is second in order depends on the initial Ta concentration in the superalloy. In superalloys A1–A6, Ti is the second most concentrated element in the MC carbides, while in superalloys A7–A9, Ta takes over.

Based on SEM-EDX results, it was shown that the addition of Ta strongly influences the relationship between the main elements that form MC carbides. The carbides were subjected to image analysis to check whether the chemical composition of the superalloy (Ta addition) influences the carbides’ size. The results are shown in Table 4. The mean size of the carbides is between 2.6 and 4.5 μm. The results indicate that there are no significant differences or relationships between the chemical composition of the carbide and its size. The carbides in all castings have a regular blocky-shaped morphology. Their non-complex shape indicates that they were formed during solidification, when a relatively high fraction of the liquid phase is present [26]. This is in line with thermodynamic simulation results, as the MC carbides are the second phase to appear directly after the γ matrix in all castings. 

**Figure 9:**
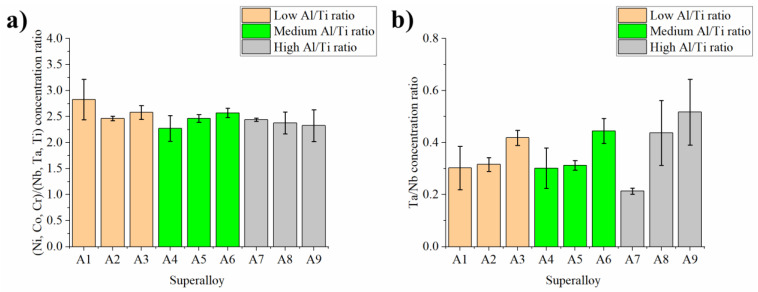
The atomic concentration relationship in the Laves phase precipitates in the interdendritic spaces: (**a**) (Ni, Co, Cr)/(Nb, Ta, Ti); (**b**) Ta/Nb, calculated based on semi-quantitative SEM-EDX measurements.

In the interdendritic spaces of all castings, there are precipitates with grey phase contrast and regular morphologies. Their amount in the microstructure is much higher than that of MC carbides. Based on SEM-EDX analysis of these precipitates, it was shown that they are rich in Ni and Nb. This unique chemical composition suggests that the Laves phase is formed with a chemical formula based on the A_2_B intermetallic compound. Position A is usually occupied by Ni, Cr, and Co, while B is occupied by Nb, Ti, and Ta. The atomic concentration relationship (Ni, Co, Cr)/(Nb, Ta, Ti) was calculated and is presented in Figure 9. This relation is within the 2.28–2.83 range. A more clear relationship is observed between the precipitates’ Ta/Nb ratio and the superalloys’ initial Ta concentration, where the increase of the latter is accompanied by an increase in the former. For Inconel 718, Baeslack et al. [27] observed that if Ta replaces Nb, Ta-rich MC carbides and Ta-rich Laves phase form. Based on microstructure observations, it was shown that the Laves phase quantities do not significantly differ between the standard superalloy and the Ta-modified one. Simulated weld HAZ experiments carried out in the Gleeble 1500 system indicated, however, a higher Laves phase liquation temperature for the Ta-modified sample (1225 °C) compared to the standard Inconel 718 alloy (1175 °C). 

**Figure 10:**
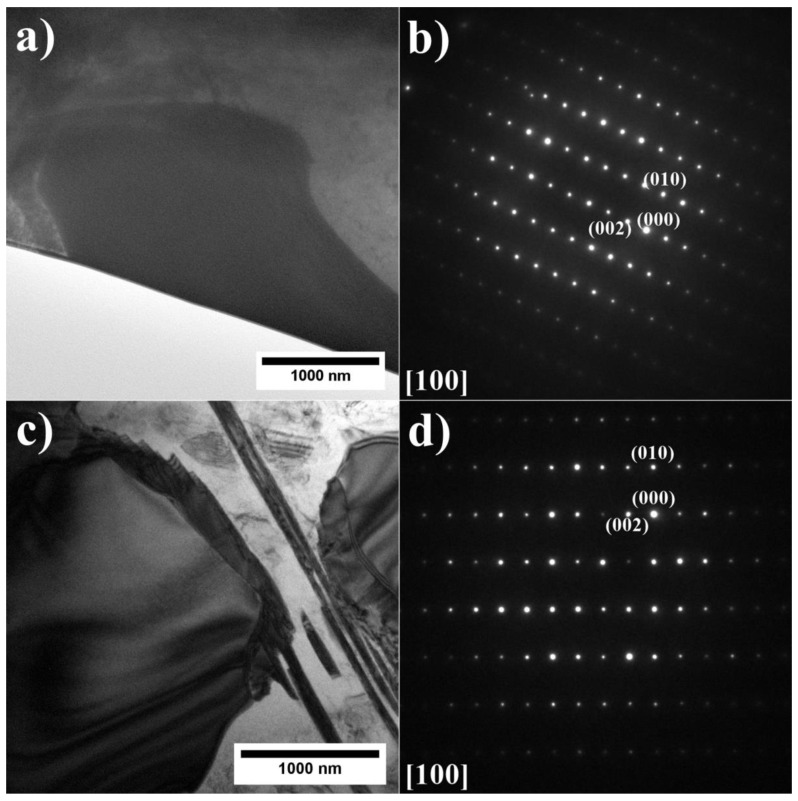
Laves phase precipitates with corresponding SAED patterns: (**a**,**b**) A3; (**c**,**d**) A6, TEM-BF.

The SAED pattern, shown in Figure 10, indicates that the crystal structure of Nb-rich precipitates is hexagonal (P6_3_/mmc), with nominal lattice parameters of a = 4.824 Å and c = 15.826 Å, consistent with the crystal structure of the Laves phase (Strukturbericht notation C36) [28]. The detected Laves phase seems to be the product of a non-equilibrium eutectic reaction L→γ + Laves occurring at the end of superalloy solidification, as predicted by Scheil solidification simulations. The solidification of superalloys begins with the formation of Nb-lean gamma dendrites which leads to enrichment of the liquid phase in Nb. Cieślak [29] stated that the solidification of Alloy 718 (and other Nb-bearing superalloys) is dominated by the segregation behavior of Nb and the precipitation of Nb-rich eutectic constituents in the form of γ/Laves and γ/MC (NbC). Radhakrishna [30] indicated that Laves phase formation requires a local niobium concentration of 10–30 wt%. The Laves phase is detrimental to the material’s mechanical properties, affecting structural integrity, and can lead to premature failure of critical components during service [30]. Their amount should be significantly decreased during solution heat treatment to avoid weak-zone microstructures between the Laves phase and the matrix interface. Additionally, plate-like precipitates are visible at the edges of the Laves phases. It has been confirmed by FIB-SEM reconstruction tomography of the as-cast 718Plus superalloy that the Laves phase/γ interfaces are favorable for η phase formation [31].

**Figure 11:**
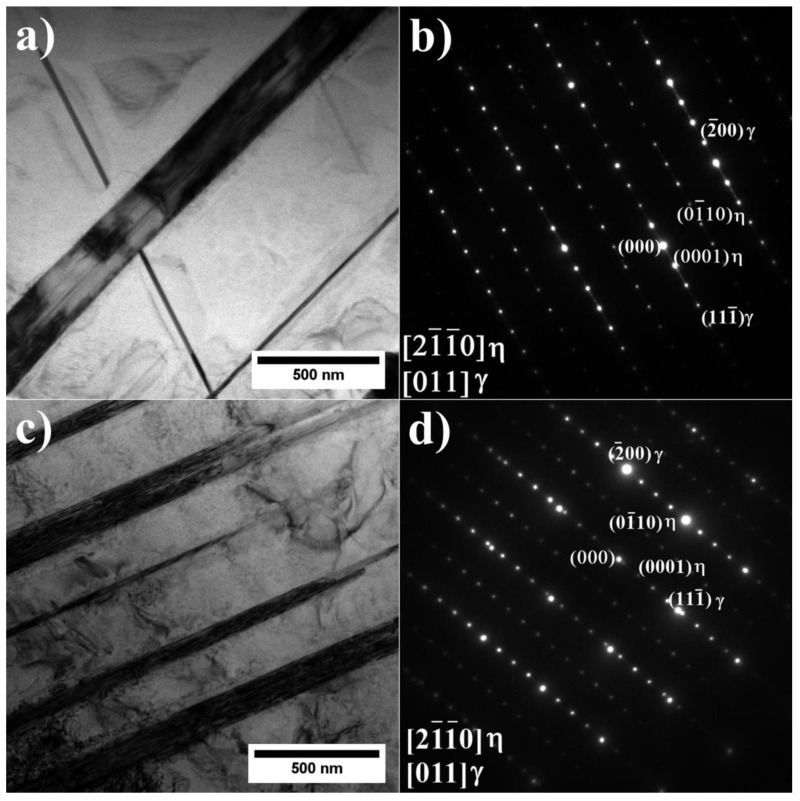
η phase precipitates with corresponding SAED patterns: (**a**,**b**) A3; (**c**,**d**) A6, TEM-BF.

Subsequently, the precipitates with plate-like morphologies, visible in variants A1–A3 and A6, were investigated using TEM. The TEM-BF images and the corresponding SAED patterns of superalloys A3 and A6 are presented in Figure 11. 

**Figure 12:**
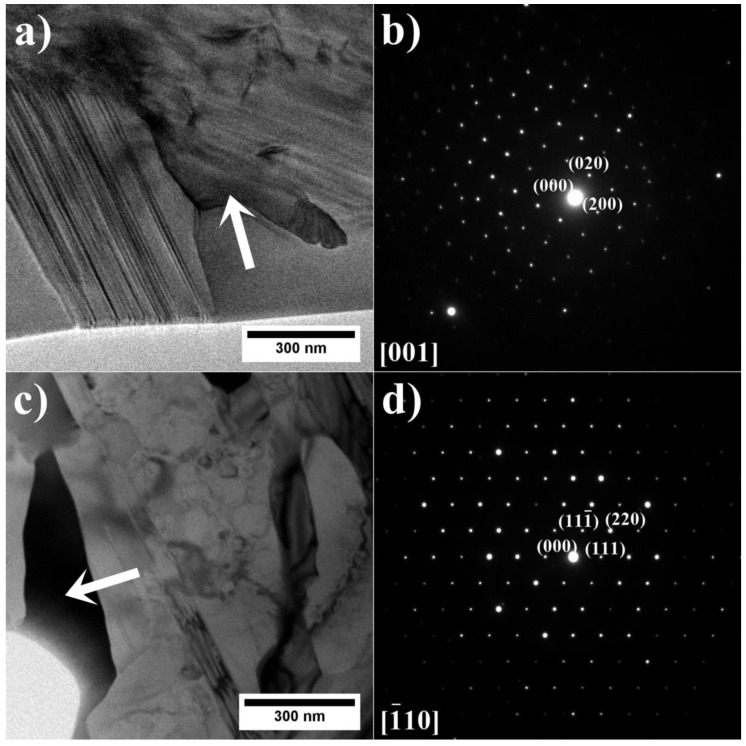
G phase precipitate with corresponding SAED patterns: (**a**,**b**) A3; (**c, d**) A6, TEM-BF.

It has been stated that those precipitates are the η (Ni_3_Ti) phase with an ordered DO_24_ hexagonal closed packed structure and nominal lattice parameter a = 5.096 Å and c = 8.3040 Å [32]. The SAED patterns confirmed that the η phase precipitates are incoherent with the γ matrix. The results suggest that large amounts of Ti and Ta favor the appearance of the η phase. It is especially visible in superalloys with a low Al/Ti ratio, as the addition of Ta causes the amount of η phase to increase compared to remelted IN740 [24]. The lack of η precipitates in superalloys A4 and A5 indicates that the increased Al concentration upon the addition of Ta at 2.0–3.0 wt% could prevent η phase formation. The results are in line with the conclusions formed by Bouse [33], who stated that the appearance of the η phase is favored by large amounts of Ti and Ta in Ni-based alloys. Seo et al. [34], based on microstructure observations of directionally solidified IN792 + Hf, indicated that the η phase was the final solidification product formed from the residual liquid after the γ-γ′ eutectic reaction. The authors stated that the concentration relationship (Ti + Ta + Hf)/Al in the residual liquid has a significant role in the nucleation of the η phase. During the solidification of eutectic γ-γ′ phase, the continual increase in the (Ti + Ta + Hf)/Al ratio in the residual liquid eventually led to the completion of the γ/γ′ eutectic reaction. Consequently, it caused η phase nucleation. According to theoretical calculations, the η phase has significant solubility for other alloying elements such as Co, Al, Cr, and W [35]. Kruk et al. [36] revealed by STEM-EDX analysis of the precipitates in the 718Plus alloy that Nb can also be present in η phase plates. Tan et al. [37] suggested that when the Ti/Al ratio in the liquid phase is higher than around 2.0, the η phase is favorable to form; however, when the ratio decreases below the key point, the eutectic γ + γ′ starts to precipitate. In the ЭК151 superalloy, the η precipitates were strongly enriched in Nb [37]. The authors found that Nb has an atom size and similar chemical properties as Ti, enabling Nb to partially substitute Ti in the crystal structure, so a ratio of (Nb, Ti)/Al equal to 2.35 was used. Considering the alloying elements’ segregation behavior and the phase transformation mechanisms, it can be stated that the additional segregation of Ti and Nb in the residual liquid phase is beneficial for η phase precipitate formation. Furthermore, the above-presented information and chemical similarities between Ta and Nb may explain the presence of the γ-γ′ eutectic and the lack of the η phase in the A4 and A5 alloys, i.e., in the alloys with a lower Ta concentration than the A6 alloy, in which the η phase is already present and the eutectic γ-γ′ is missing. Finally, near the η and Laves phase precipitates, the additional fine precipitates of the G phase are observed (Figure 12). They are complex silicides with a face-centered crystal structure and nominal lattice parameter of a = 11.2 Å [38]. The usual chemical composition is presented as (Nb, Ti)_6_(Ni, Co)_16_Si_7_.

**Figure 13:**
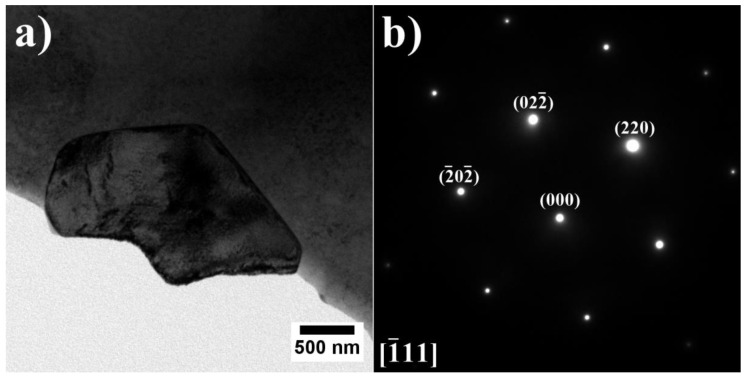
(**a**) morphology of the TiN; (**b**) corresponding SAED pattern, TEM-BF.

It was believed that the G phase was only released in austenitic steels after irradiation for a long time. Powell et al. [39] showed that the G phase could precipitate in Fe-Ni alloys, with an increased concentration of Ti or Nb, after ageing in the range of 500–850 °C. The authors suggest that this phase was misidentified as the M_6_C carbide in earlier investigations due to its similar chemical composition and structure. It was found that in Fe-Ni alloys, the G phase first precipitates on NbC carbides at the grain boundaries and, after long-term ageing, inside the grains [39]. Xie et al. [40] stated that the weight fraction of the G phase in the IN740 after standard heat treatment is equal to 0.054%, whereas after 2000 h and ageing at 760 °C, it is almost 10 times higher (0.471%). The solidification simulation predicted the existence of the G phase in superalloys A1–A6 directly in the as-cast state, which is in line with TEM observations. In the remelted IN740, precipitates with high Si concentrations, suggesting the presence of the G phase, have also been observed [24]. Ecob et al. [41] stated that the oxygen concentration influences the stability of NbC carbides compared to the G phase. The authors state that oxygen and silicon strongly segregate. Near the carbides, there is increased oxygen concentration, resulting in an uneven distribution of oxygen in the material. The simultaneous Si enrichment in some regions creates potential G phase precipitation sites. This information is important in the context of superalloy production with elevated Nb and Si concentrations, cast not under vacuum conditions, which will naturally result in higher oxygen concentrations. Additionally, precipitates with dark phase contrast were found. The SAED patterns showed that the blocky-shaped precipitates, present in all castings, were TiN with a regular crystal structure and a nominal lattice parameter of a = 4.244 Å [42] (Figure 13). The primary carbides are found to nucleate on preexisting TiN precipitates, as presented in Figure 7. 

Sobczak et al. [43], during a systematic study on the HAYNES^®^282 superalloy’s castability, carried out casting trials under non-vacuum conditions, similar to our investigation. The superalloy, which melted without deoxidizing, contained 0.38 wt% of oxygen and 0.02 wt% of nitrogen. To decrease the oxygen content, the molten superalloy was deoxidized using a NiMg alloy. It led to a three-fold decrease in oxygen (0.12 wt%). Cockcroft et al. [44] stated that the solubility of nitrogen in IN718 at 1427 °C, as determined from the solubility product of TiN, is approximately 38 ppm, which suggests that in the wrought commercial material at 60 ppm N, about 1/3 of the total N content will be present as solid TiN particles in the liquid. None of these phases were intended, with the precipitation induced by gaseous impurities (O and N), which cannot be completely removed when casting under non-vacuum conditions. Considering the thermodynamic stability of TiN, it should be underlined that it cannot be dissolved during solution heat treatment and it is insoluble up to its melting temperature. 

**Figure 14:**
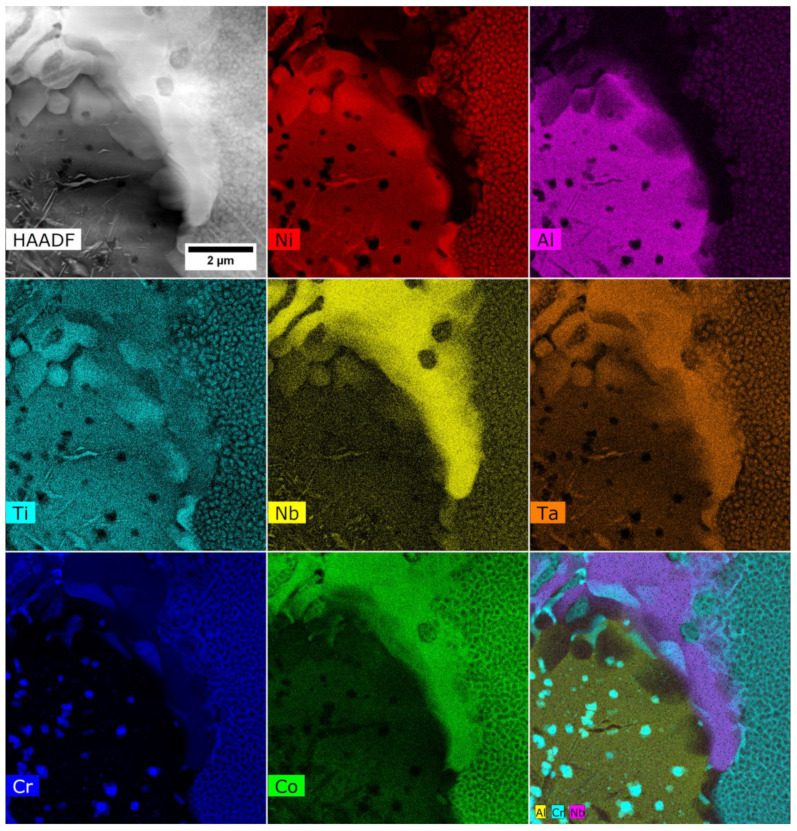
The distribution of the alloying elements in the interdendritic spaces of superalloy A9, STEM-HAADF.

In superalloys A7–A9, the interdendritic regions’ microstructure is more complex than in the case of variants A1–A6. Sample A9 was chosen as a representative variant for more detailed analysis using STEM. The STEM-EDX maps of selected alloying elements were carried out in the interdendritic region (Figure 14).

**Figure 15:**
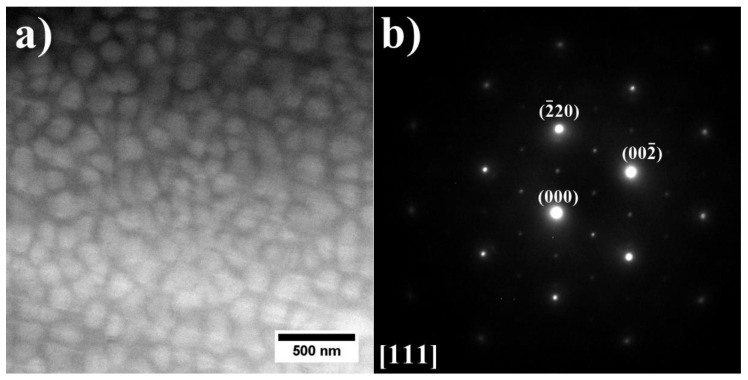
(**a**) Morphology of the γ′ precipitates in dendritic region; (**b**) corresponding SAED pattern. STEM-HAADF.

The STEM-HAADF image shows the two-phase γ + γ′ region on the right side. The areas corresponding to the fine γ′ phase precipitates are enriched in Ni, Al, Ti, and Ta, and the matrix is enriched in Co and Cr. It was confirmed by SAED that the γ matrix in this area is strengthened only by the coherent γ′ precipitates (Figure 15). In the central area, there is an island-like precipitate strongly enriched in Al. Inside, there are numerous fine precipitates with a plate-like morphology and characterized by increased Ni, Ti, Nb, and Ta concentration, as well as fine precipitates with a regular morphology, consisting of Cr. On the periphery of this island-like precipitate, there is a large Laves phase precipitate with an increased concentration of Co, Nb, Ti, and Ta. 

**Figure 16:**
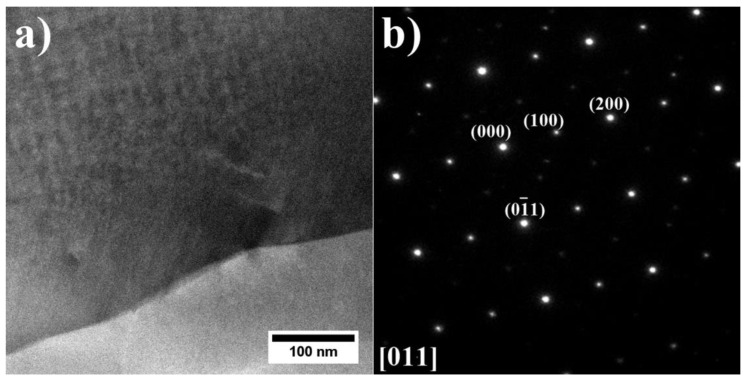
(**a**) Morphology of the β-NiAl phase island (dark part) in the interdendritic space of the superalloy A9; (**b**) corresponding SAED pattern, STEM-HAADF.

Based on the SAED of the island-like precipitate presented in Figure 16, the precipitate was identified as the β-NiAl phase, with a nominal lattice parameter of a = 2.88 Å [45].

It should be noted that despite the high Al concentration in superalloys A7–A9, the γ-γ′ eutectic did not form. Based on the Scheil calculation, the β-NiAl phase should precipitate from the liquid at relatively high temperatures. The same conclusion was formed by Song et al. [46], who observed that β-NiAl forms earlier than the γ-γ′ eutectic, which can clarify why the eutectic islands are not present in superalloys A7–A9, despite the high Al contents. Based on solidification analysis of the Ru-containing SX superalloy, Cao et al. [47] stated that the concentration of Al, Ta, and Ru in the liquid phase significantly increased during the growth of γ dendrites. EDX analysis confirmed that the main forming alloying elements of the β-NiAl phase were Ni, Al, Ta, and Ru. Therefore, the accumulation of Al, Ta, and Ru in the remaining liquid was favorable for β-NiAl formation. Tan et al. [48], based on microstructural analysis of Ru-containing SX superalloy samples obtained after DSC investigations, indicated that the cooling rate plays an important role in the β-NiAl precipitates formation. No β-NiAl precipitates were found in the microstructure created using a cooling rate of 5 °C/min. Higher cooling rates (15 °C/min and 30 °C/min) led to quick superalloy solidification, followed by less diffusion in the unsolidified interdendritic liquid phase, which resulted in the more apparent segregation of Al and Ta to interdendritic regions. The solidification induces element segregation, which results in element consumption or aggregation in the residual liquid phase. The solidification path is controlled by the change in composition. The authors also concluded that with increasing concentration of refractory alloying elements, i.e., high supersaturation, some undesirable phases would occur in Ni-based superalloys. They suggest that Ru could strongly influence the segregation behavior of Ta. It is therefore speculated that β-NiAl/Ta-rich Laves eutectics may precipitate in high-Ta-containing superalloys that are highly supersaturated during solidification [48]. 

**Figure 17:**
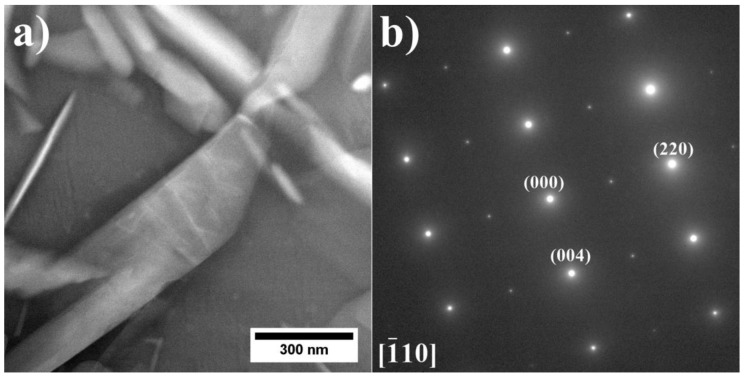
(**a**) Morphology of the η phase precipitate within β-NiAl; (**b**) SAED pattern of the η phase precipitate, STEM-HAADF.

The morphology of Ti, Ta, and Nb-rich lamellar precipitates formed inside β-NiAl is shown in Figure 17. The SAED pattern confirms the presence of the η phase with a hexagonal (DO_24_) crystal structure.

η phase precipitates were confirmed in seven out of the nine produced superalloys. The difference, however, is that in the A7–A9 superalloys, the η phase precipitates do not occur near the dual-phase γ/γ′ region or Laves phase precipitates, but are within β-NiAl. However, thermodynamic simulations predicted the formation of this phase in superalloys with a high Al/Ti concentration ratio, when the residual liquid phase is below 10%. Similar precipitates with lamellar morphologies were found inside the β-NiAl islands in Co-based superalloys [49] and Ni-based superalloys [50]. Yuan et al. [51] even observed them in the IC10 Ni_3_Al-based superalloy jointed by transient liquid phase (TLP) bonding. With the absence of Ti in both the superalloy and filler material, the monoclinic Ni_3_Ta phase was found to have precipitated via HRTEM. Pathare et al. [52], in the β-NiAl + 2 at%. Ta alloy, identified plate-like precipitates as the intermetallic compound NiAlTa with a hexagonal C14 structure, using XRD. They also stated that at least three possible ternary compounds between Ni, Al, and Ta could form: (1) NiAlTa with a hexagonal MgZn_2_ (C14)-type structure (space group P6_3_/mmc), (2) Ni_2_AlTa with a cubic Heusler alloy structure (Cu_2_AlMn-type) and nominal lattice parameter a = 5.949 Å, and (3) (Al_0_._5_Ta_0_._5_)Ni_3_ with a hexagonal Ni_3_Ti-type (DO_24_) structure, which has been confirmed in this work. 

**Figure 18:**
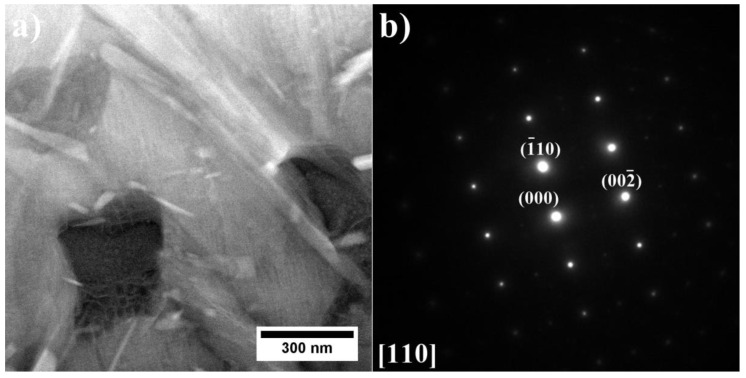
(**a**) Morphology of the α-Cr precipitates; (**b**) SAED pattern of the α-Cr precipitate, STEM-HAADF.

In addition to the plate-like precipitates, numerous fine Cr-rich (as confirmed on the EDX mapping) precipitates with a size of about 300 nm were also found inside β-NiAl (Figure 18). Chromium has limited solubility of approx. 1 at.%. in NiAl at lower temperatures. Maximum solubility additionally depends on the Ni/Al concentration ratio and is slightly greater in alloys containing less than 50% Al, and is related to the site preference of Cr for Al in NiAl [53]. Based on the SAED pattern, it is shown that the precipitates have a regular body-centered structure (BCC) with a nominal lattice parameter of a = 2.884 Å [54], which is nearly equal to the lattice parameter of the β-NiAl; therefore, only minor misfit occurs. Higher alloying levels of Cr lead to the formation of α-Cr precipitates upon slow cooling of castings. The orientation relationship between the α-Cr precipitates and the β-NiA1 matrix was determined to be cube-on-cube, i.e., <100> α {001} α//<100> β {001} β, as confirmed earlier by Cotton et al. [53]. The results of thermodynamic simulations did not show the presence of α-Cr in the as-cast A7–A9 superalloys. However, it should be noted that the simulation predicted Cr-rich phase precipitation (as shown in Figure 2 of the σ phase) at the end of solidification. The σ phase was not detected during STEM-HAADF observations. 

α-Cr has a higher melting temperature and a lower coefficient of thermal expansion compared to β-NiAl, reducing thermal stresses [53]. Based on heat-treating experiments of Ni-Al-Cr model alloys, Tian et al. [55] stated that the fine α-Cr precipitates strongly increase the hardness of NiAl thanks to the perfect lattice coherency at the α-Cr/NiAl interface. The softening that occurs after long-term annealing is caused by the loss of coherency induced by the attraction of matrix dislocations to the precipitate/matrix interface, followed by climbing around the precipitates. 

### 3.3. Analysis of the A1–A9 Superalloys Phase Transformation Temperatures during Heating

**Figure 19:**
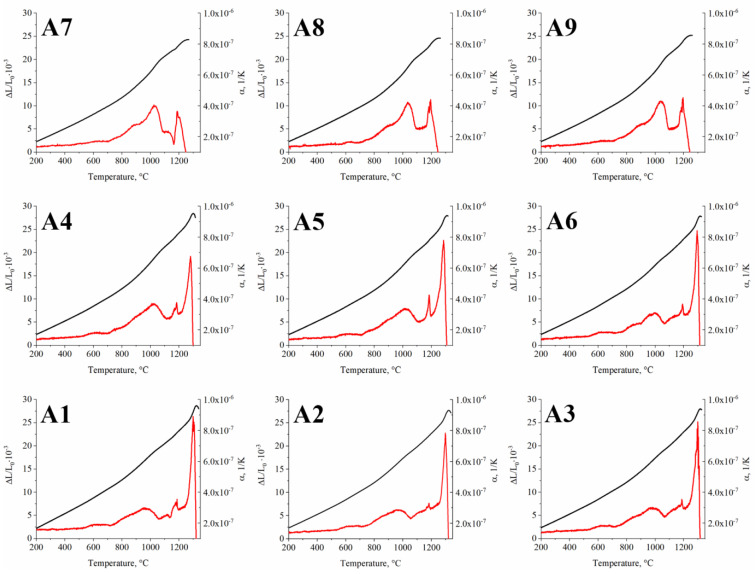
Dilatometry curves of the (**A1**–**A9**) superalloys.

The dilatometric (DIL) study was carried out to analyze phase transformations occurring in the A1–A9 superalloys during heating. The obtained curves are shown in Figure 19. The dilatation effect observed above 700 °C in all superalloys is accompanied by an increase in the coefficient of thermal expansion (CTE). The γ and γ′ phases dominate the microstructure of the A1–A9 superalloys, and it seems that changes originating from their behavior will be the most important. It could indicate that the gradual dissolution of the γ′ precipitates in the matrix causes its volume fraction to decrease. With increasing temperatures, the lattice parameters of the phases increase; however, the lattice parameter of the γ matrix increases more rapidly than that of γ′ precipitates [56,57]. Consequently, this effect would be visible on the curves as an increase in the thermal expansion coefficient, as was indeed observed. The local highest point in this dilatation effect could correspond to the temperature at which the most intense dissolution process occurs, while the end may be associated with crossing the γ′ solvus temperature. The process of γ′ dissolution takes place in a wide temperature range, which has been confirmed in many superalloys [58,59,60]. During dilatometry experiments, Trexler et al. [61] observed that the GTD111 superalloy is characterized by a rapid change in CTE above 800 °C, at which the γ′ precipitates start to dissolve. The second dilatation effect was found in the range of 1170–1190 °C for superalloys A1–A6.

Further heating significantly increased the length of the sample, accompanied by local melting. Since the effect occurs at a similar temperature for these six alloys, it must originate from the phase present in all of them. The Laves phase was found in all these alloys, characterized by a similar chemical composition and relatively high volume fraction. This effect will be discussed in greater detail in the calorimetry section. In superalloys A7–A9, Laves phase precipitation also occurs; however, the effect of their dissolution on the dilatometric curve most likely coincides with the temperature at which the sample starts to locally melt; therefore, it is not noticeable. Further heating of the samples led to a significant increase in the coefficient of thermal expansion, which is most likely related to the initiation of bulk sample melting.

**Figure 20:**
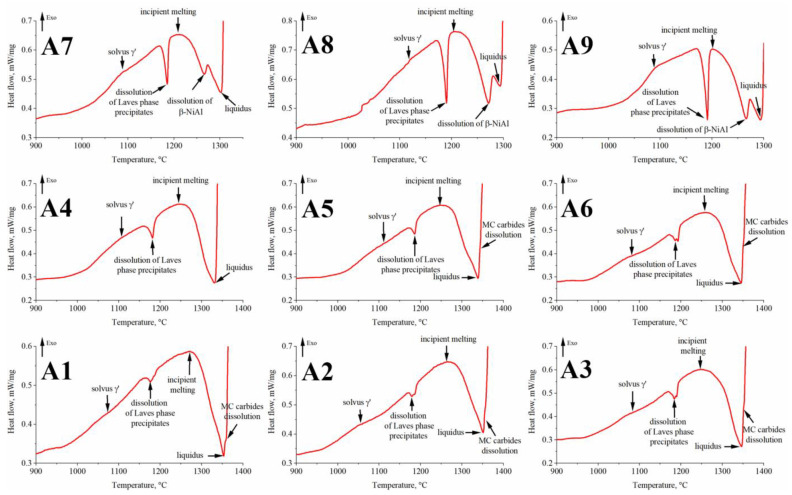
Representative DSC heating curves with a heating rate of 10 K min^−1^ for the as-cast superalloys.

The phase transformation temperatures in the as-cast superalloys were also analyzed via DSC (Figure 20) and the temperatures are summarized in Table 5. Knowledge of γ′ solvus temperatures is often important in controlling solution heat treatment temperatures before precipitation hardening. Moreover, the solvus temperature of the γ′ phase is closely related to the temperature-bearing capacity of Ni-based superalloys. Therefore it is crucial to investigate the effect of alloying elements on the solvus temperature when designing novel materials. 

In low Al/Ti ratio superalloys, the solvus temperature is in the range of approx. 1055–1084 °C. In the second group, these values are slightly higher, i.e., 1080–1111 °C. This indicates that the Al concentration has a greater influence on the solvus temperature than Ta. This is most likely due to Ta being a significant element in other phases, like MC carbides and the η phase. In superalloys with a high Al/Ti concentration ratio, the γ′ solvus temperature is 1087–1117 °C. However, it should be noted that a large amount of Al forms β-NiAl precipitates, which are still stable at this temperature. The values of γ′ solvus determined by means of the DSC curves are very close to the end temperature of the dilatation effects. The γ′ solvus temperature of commercial superalloys IN740 (Ti = 1.8 wt%, Al = 0.9 wt%) and IN740H (Ti = 1.35 wt%, Al = 1.35 wt%) is lower, 860 °C and 975 °C, respectively, as determined by Xie [40] via thermodynamic simulations. It results from the lower amount of γ′-formers as compared to the A1–A9 superalloys. A very clear endothermic effect is observed in all superalloys above 1170 °C. Despite the differences in chemical composition, the temperature range in which this effect is observed is relatively narrow, i.e., 1176–1193 °C. In each of the individual groups (low, medium, and high Al/Ti ratio), the peak temperature increases with Ta contents. The values recorded on the DSC curves are very similar to those that correspond to the dilatation effect on the DIL curves. As previously, this effect most likely comes from the Laves phase. A similar endothermic peak within the temperature range of 1162–1190 °C was observed by Cao et al. [62] in the differential thermal analysis (DTA) thermograms of Alloy 718 in as-cast condition. The effect was assigned to the Laves phase eutectic reaction. Directly after initial melting, the liquation of the existing Laves eutectic and matrix was reported to rapidly increase with temperature. This phenomenon was responsible for the endothermic process after the Laves peak on the thermograms. The authors also investigated how earlier superalloy annealing influences the latter melting process of Alloy 718. They proved that the incipient melting temperature of a fully homogenized (T = 1190 °C, t = 48 h) superalloy was higher, approx. 1230 °C, which is the solidus temperature of Alloy 718. These results suggest that the solution heat treatment of the A1–A9 superalloys should include a step before the final temperature to prevent the local melting of Laves phase. 

Further heating led to crossing the solidus temperature of the superalloys. In the A1–A3 superalloys, it is in the range of 1224–1263 °C. In the A4–A6 variants, it increases with the Ta concentration from 1245 °C to 1258 °C. In superalloys A7–A9, the solidus temperature decreased with increasing Ta contents from 1211 °C to 1201 °C. The β-NiAl phase, also present in the A7–A9 superalloys, dissolves at much higher temperatures. An inflection is registered in the range of 1266–1272 °C. During DSC experiments of as-cast superalloy SX, Song et al. [46] registered incipient melting at 1321 °C and β-NiAl dissolution at 1346 °C, when a significant amount of liquid phase was present. The high melting point of the β-NiAl phase can induce impediments in homogenizing heat treatment. Based on long-term annealing experiments, Song et al. [45] indicated that during high-temperature exposure, Ni and Al diffuse to the surrounding γ and γ′ phases, and suggested that β-NiAl can be eliminated through exposure at 1100 °C for 1000 h. 

Superalloys with a low Al/Ti ratio have the highest liquidus temperatures, whereas it is lower for each of the following two groups due to higher Al concentrations. As the Ta concentration increases, it begins to decrease in superalloys A1–A3 and A7–A9. The lower liquidus temperature in superalloys A4 and A5 in relation to A6 may result from the earlier dissolution of γ/γ′ eutectic islands and, in turn, the enrichment of the liquid in Al. The last recorded effect originates from the dissolution of MC carbides. In the A4 and A7–A9 superalloys, no thermal effects were recorded. However, this is not due to the lack of MC carbides, the presence of which was confirmed during microstructure observations. Liu et al. [63], who investigated the influence of temperature on the homogenization of melted Inconel 738LC, showed that MC carbides could survive at a temperature much higher than liquidus. At 1550 °C, the MC carbides did not completely dissolve in the liquid after about 5–10 min. For comparison, the liquidus temperature of Inconel 738LC superalloy is lower, 1336–1342 °C [64].

## 4. Conclusions

Based on the results and observations of as-cast model A1–A9 superalloys, characterized by various Al/Ti ratios and Ta concentrations, the following conclusions are proposed:

- The Scheil solidification simulation showed the formation of the γ, γ′, MC carbides, and Laves phase in all castings. Additionally, β-NiAl was predicted in superalloys with high Al/Ti ratios. G phase precipitates could form in the final stage of solidification in superalloys with low and medium Al/Ti ratios.
- For all castings, the XRD spectra revealed peaks corresponding to γ matrix, γ′ precipitates, and MC. The β-NiAl phase is present in castings characterized by high Al/Ti ratios.
- As verified by SEM and TEM, the microstructure of alloys A1–A9 consists of a γ matrix, γ′ phase, (Nb, Ti, Ta)C carbides, and Laves phase precipitates. The alloys with low and medium Al/Ti ratios, except variants A4 and A5, were additionally strengthened by plate-like η phase precipitates.
- It was shown via SEM-EDX analysis that the initial Ta concentration in the superalloy has a strong influence on the MC carbides’ composition, as it can partially replace Nb and Ti. The Ta/Nb and Ta/Ti concentration relationship in carbides increases with Ta content in the superalloy.
- The high initial Al/Ti concentration ratio in the A7–A9 superalloys led to the formation of the β-NiAl phase, strengthened locally by η phase and α-Cr precipitates.
- On the DIL curves of all alloys, dilatation effects originating from γ′ precipitates are observed. In the A7–A9 superalloys, the effect from Laves phase dissolution was registered right before sample melting.
- Based on the DSC curves, the γ′ solvus temperature in alloys with low and medium Al/Ti ratios was in the range of 1055–1084 °C and 1080–1111 °C, respectively. The liquidus temperature decreased with the increase in Al/Ti ratio.

## Figures and Tables

**Table 1:** IN740 modification procedures, wt%.

Ta Concentration	Al/Ti Concentration Ratio		
	Low: 0.7 (±0.10)	Medium: 1.5 (±0.15)	High: 3.4 (±0.20)
Low: 2.0 (±0.10)	A1	A4	A7
Medium: 3.0 (±0.15)	A2	A5	A8
High: 4.0 (±0.20)	A3	A6	A9

**Table 2:** A1–A9 superalloy chemical composition, wt%.

Alloy	Cr	Co	Al	Ti	Nb	Fe	Mo	Mn	Si	C	Ta	Ni
A1	22.34	19.96	1.30	1.72	1.80	0.62	0.43	0.30	0.59	0.03	2.00	Balance
A2	22.46	19.91	1.28	1.78	1.92	0.61	0.43	0.30	0.60	0.02	3.00	
A3	22.41	19.91	1.31	1.68	1.88	0.62	0.44	0.30	0.61	0.02	4.00	
A4	22.84	19.71	2.80	1.82	1.92	0.63	0.42	0.29	0.59	0.03	2.00	
A5	22.74	19.69	2.82	1.76	1.80	0.64	0.43	0.30	0.60	0.03	3.00	
A6	22.28	19.85	2.75	1.83	1.94	0.56	0.43	0.22	0.56	0.02	4.00	
A7	21.76	18.85	6.06	1.81	1.88	0.63	0.42	0.29	0.60	0.03	2.00	
A8	21.20	19.05	6.34	1.76	1.82	0.61	0.40	0.28	0.56	0.03	3.00	
A9	21.43	18.65	6.12	1.84	1.86	0.62	0.42	0.29	0.60	0.03	4.00	

**Table 3:** γ′ precipitates size in the dendritic regions.

Parameter/Casting	A1	A2	A3	A4	A5	A6	A7	A8	A9
Mean size of the γ′ precipitates, nm	26 (±3)	31 (±4)	46 (±6)	99 (±10)	76 (±9)	41 (±4)	110 (±16)	112 (±25)	134 (±18)
Circularity, ξ	0.96 (±0.04)	0.93 (±0.03)	0.93 (±0.02)	0.90 (±0.02)	0.91 (±0.04)	0.94 (±0.02)	0.89 (±0.02)	0.88 (±0.03)	0.87 (±0.03)

**Table 4:** Mean size of the MC carbides in A1–A9 superalloy castings.

Superalloy	Mean Size, μm	Standard Deviation, μm
A1	3.1	1.2
A2	3.8	1.2
A3	3.1	1.6
A4	3.5	1.5
A5	3.0	0.7
A6	2.7	0.7
A7	4.5	1.3
A8	2.6	0.9
A9	3.1	0.9

**Table 5:** Phase transformation temperatures of the A1–A9 superalloys registered during DSC heating.

Alloy	γ′ solvus	Dissolution of Laves Phase	Incipient Melting (Solidus)	β-NiAl Dissolution	Liquidus	MC Carbides Dissolution
A1	1072	1176	1224	not registered	1353	1357
A2	1055	1179	1263		1351	1354
A3	1084	1184	1247		1346	1350
A4	1106	1182	1245		1330	not registered
A5	1111	1185	1250		1339	1343
A6	1080	1193	1258		1345	1350
A7	1089	1185	1211	1267	1302	not registered
A8	1117	1189	1208	1272	1293	
A9	1087	1190	1201	1266	1292	

## Data Availability

Not applicable.
